# Papulonodular mucinosis, Guillain-Barré syndrome and nephrotic syndrome in a patient with systemic lupus erythematosus: a case report

**DOI:** 10.1186/s12882-017-0458-0

**Published:** 2017-02-01

**Authors:** Xiaole Su, Xi Qiao, Jing Li, Lifang Gao, Chen Wang, Lihua Wang

**Affiliations:** 1 Renal Division, Shanxi Medical University Second Hospital, Shanxi Kidney Disease Institute, No.382, Wuyi Road, Xinghualing Distirct, Taiyuan, Shanxi Province China; 2 Pathology Division, Shanxi Medical University Second Hospital, Shanxi Province Kidney Pathology Centre, No.382, Wuyi Road, Xinghualing Distirct, Taiyuan, Shanxi Province China

**Keywords:** Nephrotic syndrome, Papulonodular mucinosis, Guillain-Barré syndrome, Systemic lupus erythematosus

## Abstract

**Background:**

Awareness of the spectrum of clinical manifestations of systemic lupus erythematosus (SLE), especially uncommon changes, is essential for diagnosis and effective management of patients.

**Case presentation:**

A 26-year-old Chinese man with SLE initially manifested cutaneous papulonodular mucinosis and developed acute Guillain-Barré syndrome and class V lupus nephritis 2 years later. His cutaneous nodules had not been idententified for 2 years and were resected by surgical procedures twice until SLE was diagnosed. The kidney biopsy revealed class V lupus nephritis. The patient responded well to a short course of intravenous immunoglobulins and his muscle strength almost completely recovered. So far, he has undergone five cycles of cyclophosphamide combined with hydroxychloroquine and tapering prednisone, resulting in partial remission of lupus nephritis and disappearance of hypocomplementemia.

**Conclusion:**

We reported a rare case of male patient with SLE with manifestation of class V lupus nephritis, Guillain-Barré syndrome and papulonodular mucinosis.

## Background

Systemic lupus erythematosus (SLE) is a complex multi-system autoimmune disease presenting with a wide spectrum of clinical and immunological abnormalities [1]. Skin involvement occurs in more than 85% of lupus erythematosus (LE) cases, and the four classic presentations include malar rash, discoid rash, photosensitivity and oral mucosal lesions [2]. Lupus-related polyneuropathy have been reported in 10–20% of patients with SLE [3]. Many different types of neuropsychiatric symptoms occur in patients with SLE. The kidney is also one of most frequently involved organs. The reported incidence of clinically important kidney diseases in systemic lupus varies widely, range from 12 to 69% [4]. However, patients with SLE may present with an array of manifestations not limited to these common presentations, such as Guillan Barré syndrome (GBS), papulonodular mucinosis (PNM) and class V nephritis which are all rarer associations in SLE than describing in general about neurological, mucocutaneous manifestations and renal involvement in SLE. Awareness of the spectrum of clinical manifestations of SLE, especially uncommon changes, is essential for diagnosis and effective management of patients. We describe a rare case of SLE in a patient who initially had cutaneous PNM and had not been identified for 2 years until GBS and class V lupus nephritis were detected.

## Case presentation

A 26-year-old Chinese man presented with progressive numbness, weakness and paresthesia of the extremities. One month before admission, he began to complain of mild symmetrical numbness, weakness and paresthesia of limbs (with “socks and gloves” distribution). Simultaneously, he was found to have transient facial erythema after exposed to ultraviolet light, which disappeared spontaneously without treatment. One week before admission, his numbness and weakness in lower extremities progressed to limit his ambulation. His medical past history was notable for isolated nodular masses twice without pain in the cheeks below the left and right earlobe in 2013 and 2014, which were 4 × 5 cm (left) in 2013 and 3 × 3 cm (right) in 2014. They were both resected with a pathological diagnosis of “nodular fasciitis” in his local hospital. He also complained of moderate fatigue and alopecia for 2 years. There was no family history of other connective diseases. The physical examination showed that there was motor weakness with a power of 2 of 5 in lower extremities and 3 of 5 in upper extremities. The sensory examination was normal. Deep tendon reflexes were decreased and plantar reflexes were flexor. The rest of the examination was unremarkable.

Laboratory assessment indicated a erythrocyte sedimentation rate (ESR) of 79 mm/h. Urinalysis showed protein in 3+, urinary sediment 0 to 2 red cells per 10*40 field and no granular casts. Urine protein excretion was 7.00 g/24 h and serum albumin was 1.8 g/dL. The results of the blood cell count and hepatic function tests were normal. The levels of serum creatinine, and urea nitrogen were normal, respectively, 68 μmol/L (range 57–97 μmol/L) and 3.8 mmol/L (2.6–7.5 mmol/L). Immunological screening revealed an antinuclear antibody titer of 1:320, a speckled pattern and a double-stranded DNA antibody titer of 1:20 positive. The rest of the autoantibody profiles, including anti-smith, anti-ribonucleoprotein and antineutrophil cytoplasmic antibodies, were negative. Serum C3 and C4 complement factors were low, respectively, 0.43 g/L (range 0.65–1.65 g/L) and 0.07 g/L (range 0.16–0.6 g/L). The cerebrospinal fluid examination showed an increased protein concentration of 1.09 g/L and immunoglobulin (Ig) G level of 0.28 g/L with a normal number of cells (white cell count was zero). Electromyography revealed a severe demyelinating polyneuropathy and a muscle biopsy revealed neurogenic muscle damage.

**Fig. 1:**
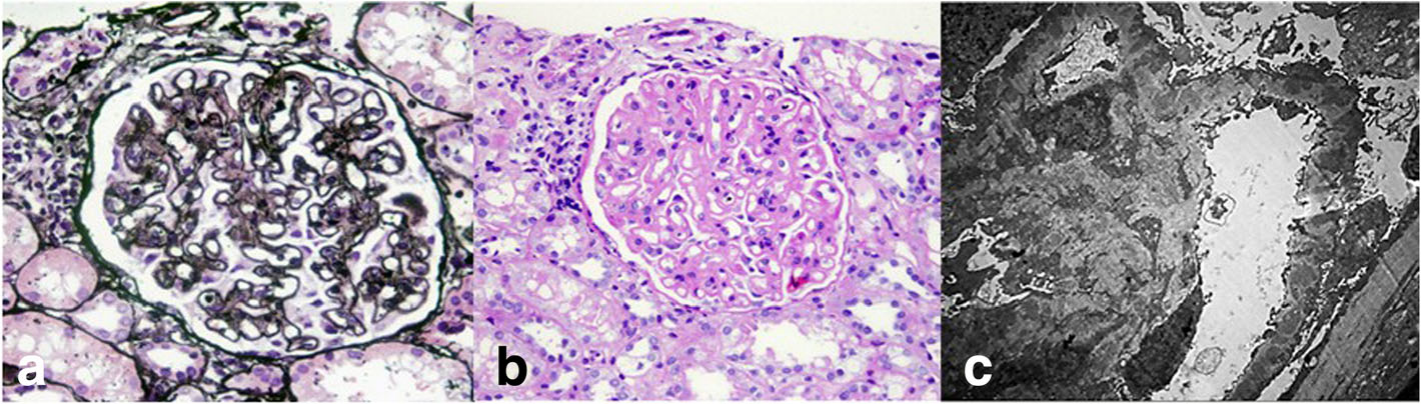
Renal histopathological findings. Light micrographs of a glomerulus show diffuse thickening of the glomerular basement membrane (Periodic acid-silver metheramine stain; 400×) (**a**) with mesangial hypercellularity and mesangial matrix expansion (Schiff periodic acid shiff stain; 400×) (**b**); electron microscopic analyses show numerous subepithelial and mesangial electron-dense deposits, as well as deposits within the glomerular basement membrane with extensive fusion of foot processes (5000×) (**c**)

**Fig. 2:**
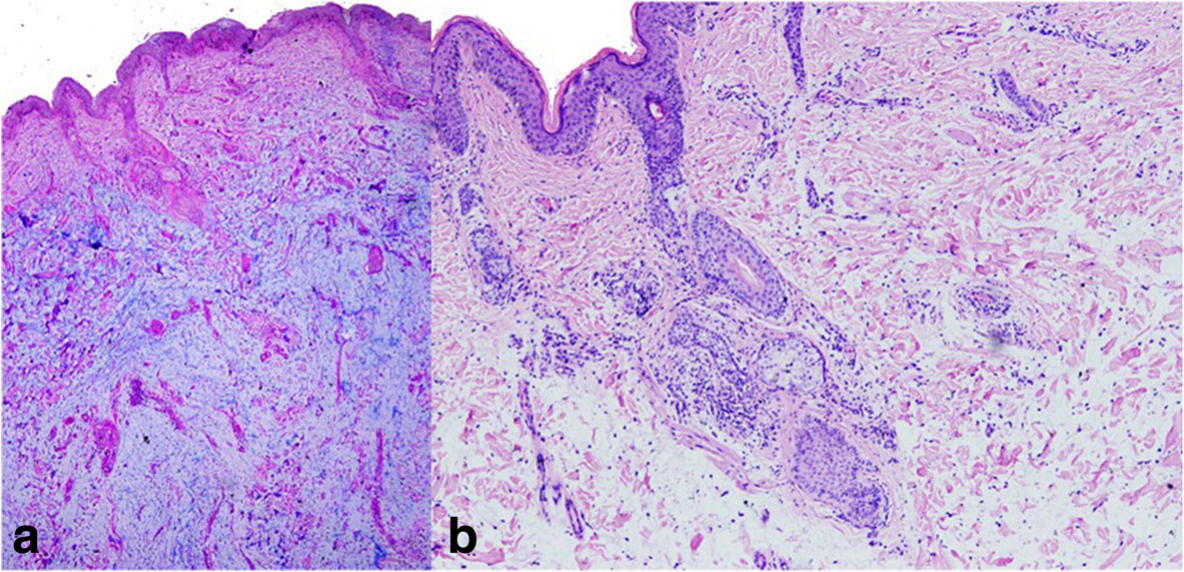
Cutaneous histopathological findings. Light micrographs of a nodular mass biopsy specimen show diffuse mucinous material deposited in the dermis (Alcian blue-schiff periodic acid shiff stain; 40×) (**a**) with a lymphocytic and plasmacytic infiltrate (confirmed by immunohistochemistry) in perivascular and periadnexal tissue (Hematoxylin and eosin stain; 100×) (**b**)

Kidney biopsy was performed and revealed diffuse thickening of the glomerular basement membrane with “spike” formation, mesangial hypercellularity and mesangial matrix expansion (Fig. 1). Immunofluorescence revealed 1 to 3+ global granular capillary wall and mesangial staining for IgG, IgA, IgM, C3, C1q, and fibrin-fibrinogen, which gave the characteristic “full house” immunofluorescence for lupus nephritis. The electron microscopic analyses showed numerous subepithelial and mesangial electron-dense deposits, as well as deposits within the glomerular basement membrane with extensive fusion of foot processes. The renal biopsy diagnosis was secondary membranous nephropathy and suspected lupus nephritis. Two biopsy specimens of nodular masses from the cheeks were rechecked by our institution and revealed similar features (Fig. 2); namely, diffuse mucinous material deposited in the dermis with a moderate lymphocytic and plasmacytic infiltrate in perivascular and periadnexal tissue. Immunohistochemistry revealed IgG deposition at the dermoepidermal junction. These findings were characteristic of cutaneous PNM.

The patient was diagnosed as having SLE with manifestation of class V lupus nephritis, GBS and PNM. He received intravenous immunoglobulin therapy at 0.4 g/kg/d for 5 days, then hydroxychloroquine 200 mg twice daily, and an initial 1 mg/kg daily dose of prednisone and 0.8 g cyclophosphamide once a month. At discharge, he had partial recovery of his muscle strength in lower limbs to 3/5 and upper limbs to 4+/5. After five cycles of cyclophosphamide, his albumin level improved from 1.8 g/dl to 3.0 g/dl, urinary protein level fell from 7 g/24 h to 2.3 g/24 h, ESR was 17 mm/h and levels of C3, C4 increased to the normal range. He was able to walk unassisted and had full recovery of his muscle strength in all four limbs. Until now, he is still in follow-up treatment and has not developed muscle weakness, skin involvement or any other systemic manifestations of lupus.

## Discussion

We describe here a SLE patient with PNM, GBS and class V lupus nephritis. His skin nodules had not been identified for 2 years until SLE was diagnosed. A combined treatment with intravenous immunoglobulin, tapering prednisone, hydroxychloroquine, and cyclophosphamide successfully recovered his muscle strength and also induced the partial remission of lupus nephritis.

Cutaneous mucinosis is a group of disorders characterized by a prominent accumulation of mucin in the interstitial spaces of the dermis, which are classified as primary and secondary [5]. Some of the secondary mucinosis are associated with SLE, and may even be its initial manifestation [5, 6]. PNM, also known as cutaneous lupus mucinosis, is an uncommon disorder that typically presents as asymptomatic skin-colored or pink-to-red nodules or/and papules varying in size on the upper parts including arms, head, neck, the back and the chest [5]. There are rare reported cases of PNM occurring on the lower extremities [7]. According to the literature [8], the sex ratio of PNM is 11:13 (men:women). Considering the female preponderance of SLE, 1:9 (men:women), the male incidence of PNM is extremely high, and our patient is male.

In patients with PNM, histopathologic examination reveals abundant deposits of mucin located primarily in the papillary and mid-dermis, interspersed among collagen bundles. There may be a slight perivascular lymphocytic infiltrate in the papillary dermis. The epidermal changes in PNM are not the typical changes as seen in specific LE lesions. Typical linear or granular deposits of immunoglobulins (IgG and IgM) and C3 at the dermoepidermal junction can be seen and are sufficient to constitute a positive lupus band test [5, 9]. These deposits are very similar to those seen in our patient.

The clinical manifestations of this patient with neuropsychiatric symptoms (GBS), skin (PNM and malar rash) and renal involvement (class V lupus nephritis), and laboratory assessments with hypocomplementemia, positive antinuclear and double-stranded DNA antibody, satisfied 5 of 11 American College of Rheumatology (ACR) criteria, and 3 clinical and 3 immunologic criteria of the Systemic Lupus International Collaborating Clinics (SLICC) criteria; accordingly, the patient was diagnosed as having SLE. Nevertheless, he presented with PNM twice, one and two years before admission, which probably corresponded to the initial presenting signs of SLE. PNM may be in isolation or in combination with typical lesions of LE. In one series, 80% of LE patients with PNM had either joint or renal involvement [10], which underlines high frequency of kidney involvement might occur in patients with SLE and having PNM. Our patient had class V lupus nephritis confirmed by biopsy, which is much rarer compared to Class III or IV nephritis in SLE.

Simultaneously, due to progressive symmetric numbness, weakness and hyporeflexia of limbs and albuminocytologic dissociation of cerebrospinal fluid, he was diagnosed as having GBS. To the best of our knowledge, this case is the first reported case of PNM to be associated with GBS and lupus nephritis. GBS is an acute, ascending, inflammatory, demyelinating polyneuropathy and constitutes part of the ACR neuropsychiatric LE case definition [11]. A retrospective series of 2097 patients with SLE in a 25-year study period showed only one patient to have GBS [12]. By contrast, a study by Leneman has reported seven patients out of a series of 1100 with GBS who had SLE [13]. GBS is therefore considered to be extraordinarily rare in SLE [12].

The response of PNM to treatment has been found to be variable. In some cases, PNM lesions may remit spontaneously. Treatments that are used for LE, including antimalarials, or topical or oral corticosteroids have also been used for PNM with variable results [14]. Injection of lesions with hyaluronidase has also been reported to be effective [15]. In this case, improvement of neurological and renal manifestations with intravenous immunoglobulin, prednisone and cyclophosphamide suggests an autoimmune pathogenesis. We speculate that this may represent a single or several cross-reactive autoantibodies in neural and renal tissue. Furthermore, the pathogenesis of PNM is also largely unknown. In our case, PNM recurred 1 year after the first surgical resection of a nodule, and then the patient underwent the second resection. To date, PNM has not relapsed but we do not know if this is related to the drug treatment. Presumably, common immunological pathways could tie together these three manifestations.

## Conclusions

In conclusion, the present case is the first case report of PNM combined with GBS and class V lupus nephritis in the background of SLE. In patients with PNM, especially those with neurological symptoms and renal involvement, clinicians should have a high index of suspicion for the presence of systemic diseases, especially SLE.

## Abbreviations

ESR: Erythrocyte sedimentation rate
GBS: Guillain-Barré syndrome
Ig: Immunoglobulin
LE: Lupus erythematosus
PNM: Papulonodular mucinosis
SLE: Systemic lupus erythematosus

## References

[1] Mills JA (1994). Systemic lupus erythematosus. N Engl J Med.

[2] Uva L, Miguel D, Pinheiro C, Freitas JP, Marques Gomes M, Filipe P (2012). Cutaneous manifestations of systemic lupus erythematosus. Autoimmune Dis.

[3] Levin KH (2004). Variants and mimics of Guillain Barre Syndrome. Neurologist.

[4] Kidney Disease: Improving Global Outcomes (KDIGO) Glomerulonephritis Work Group: KDIGO Clinical Practice Guideline for Glomerulonephritis. Kidney Int Suppl. 2012; 2: 139–274.

[5] Rongioletti F, Rebora A (2001). Cutaneous mucinoses: microscopic criteria for diagnosis. Am J Dermatopathol.

[6] Mascaro JM, Herrero C, Hausmann G (1997). Uncommon cutaneous manifestations of lupus erythematosus. Lupus.

[7] Shekari AM, Ghiasi M, Ghasemi E, Kani ZA (2009). Papulonodular mucinosis indicating systemic lupus erythematosus. Clin Exp Dermatol.

[8] Kanda N, Tsuchida T, Watanabe T, Tamaki K (1997). Cutaneous lupus mucinosis: a review of our cases and the possible pathogenesis. J Cutan Pathol.

[9] Rongioletti F, Parodi A, Rebora A (1990). Papular and nodular mucinosis as a sign of lupus erythematosus. Dermatologica.

[10] Elkeeb L, Spicknall KE, Mutasim DF (2014). Nodular cutaneous mucinosis associated with systemic lupus erythematosus. Int J Dermatol.

[11] The American College of Rheumatology nomenclature and case definitions for neuropsychiatric lupus syndromes. Arthritis Rheum. 1999; 42: 599–608. 10.1002/1529-0131(199904)42:4<599::AID-ANR2>3.0.CO;2-F 10211873

[12] Oomatia A, Fang H, Petri M, Birnbaum J (2014). Peripheral neuropathies in systemic lupus erythematosus: clinical features, disease associations, and immunologic characteristics evaluated over a twenty-five-year study period. Arthritis Rheum.

[13] Leneman F (1966). The Guillain-Barre syndrome. Definition, etiology, and review of 1,100 cases. Arch Intern Med.

[14] Lowe L, Rapini RP, Golitz LE, Johnson TM (1992). Papulonodular dermal mucinosis in lupus erythematosus. J Am Acad Dermatol.

[15] Maruyama M, Miyauchi S, Hashimoto K (1997). Massive cutaneous mucinosis associated with systemic lupus erythematosus. Br J Dermatol.

